# Models of Dendritic Cells to Assess Skin Sensitization

**DOI:** 10.3389/ftox.2022.851017

**Published:** 2022-03-18

**Authors:** Kévin Hardonnière, Natacha Szely, Zeina El Ali, Marc Pallardy, Saadia Kerdine-Römer

**Affiliations:** Université Paris-Saclay, Inserm, Inflammation, Microbiome and Immunosurveillance, Châtenay-Malabry, France

**Keywords:** ACD, DCs, skin, sensitization, Mo-DCs, CD34-DCs, BM-DCs

## Abstract

Allergic contact dermatitis (ACD) is a complex skin pathology occurring in reaction against environmental substances found in the workplace (cement, hair dyes, textile dyes), in the private environment (e.g., household products, cosmetic ingredients), or following skin exposure to drugs. Many cells are involved in the initiation of ACD during the sensitization phase. The four key events (KE) of skin sensitization AOP are covalent binding to skin proteins (KE1), keratinocyte activation (KE2), activation of DCs (KE3), and T-cell activation and proliferation (KE4), leading to the adverse outcome of ACD. Dendritic cells (DCs) are thus playing a key role in ACD pathophysiology. Indeed, in the presence of chemical sensitizers, DCs migrate from the skin to the draining lymph nodes and present peptide-chemical conjugates to T cells, leading to their activation and proliferation. *In vitro* methods have been actively developed to assess the activation of DCs by chemicals to establish a reliable *in vitro* sensitization test. Therefore, this review will detail the most used methods and protocols to develop DC models *in vitro*. Three different models of DCs will be addressed: 1) DCs derived from Cord Blood (CD34-DCs), 2) DCs derived from Monocytes (Mo-DCs), and 3) DCs derived from mice Bone-Marrow (BM-DCs). In addition, a model of exposition to contact sensitizers to assess KE3 of skin sensitization will be detailed for each of the models presented.

## Introduction

Skin sensitization has been described in an adverse outcome pathway (AOP) with defined key events (KEs) aiming to increase its mechanistic understanding (OECD) (Natsch, 2013; Rovida et al., 2015). Skin sensitization is the first step of Allergic Contact Dermatitis (ACD), a common inflammatory skin disease in humans with a prevalence of 15–20% in the general population (Honda et al., 2013).

ACD is classified as a delayed hypersensitivity reaction that occurs after skin exposure to contact sensitizers (CS) or contact allergens, also known as haptens due to their protein-binding properties, involving dendritic cells (DCs) (Martin, 2011). ACD is composed of two phases: the sensitization phase, clinically silent, and the inflammatory elicitation phase. The sensitization phase occurs when the CS penetrates the epidermis, whereas the elicitation phase is responsible for the recruitment of specific T cells at the site of chemical application. It occurs upon a reexposure with the same chemical or, in case of cross-reactions with chemicals having a similar structure (Martin, 2011; Christensen and Haase, 2012; Popov et al., 2012). DCs play a significant role in initiating the chemical-specific primary immune response during the initial phase of sensitization.

DCs are responsible for the initiation of adaptive immune responses and hence function as the ‘sentinels’ of the immune system. Epidermal Langerhans cells (LCs) and dermal DCs (dDCs) capture the hapten-protein conjugates and migrate to skin-draining lymph nodes (LN) under the influence of inflammatory cytokines like IL-1β and TNF-α, generated in the skin following CS application. In draining LN, DCs present haptenized peptides bound to MHC-I or MHC-II molecules to naïve hapten-specific CD8 and CD4 T cells, respectively, leading to their activation and proliferation (Popov et al., 2012). This leads to the generation of skin-homing CD8+T cytotoxic (Tc) 1/Tc17 and CD4 ^+^T helper-type (Th) 1/Th17 effector T cells that enter into the blood circulation (Zhao et al., 2009; Martin, 2012). Upon reexposure of the skin, the CS triggers a cascade of events resulting in the infiltration of monocytes, neutrophils, and effector T cells. Specific T cells recruited in the skin produce Th1 and Th17 cytokines that include IFN-γ, IL-2, and IL-17, 24 h after the challenge (Martin, 2011; Peiser et al., 2012).

**FIGURE 1:**
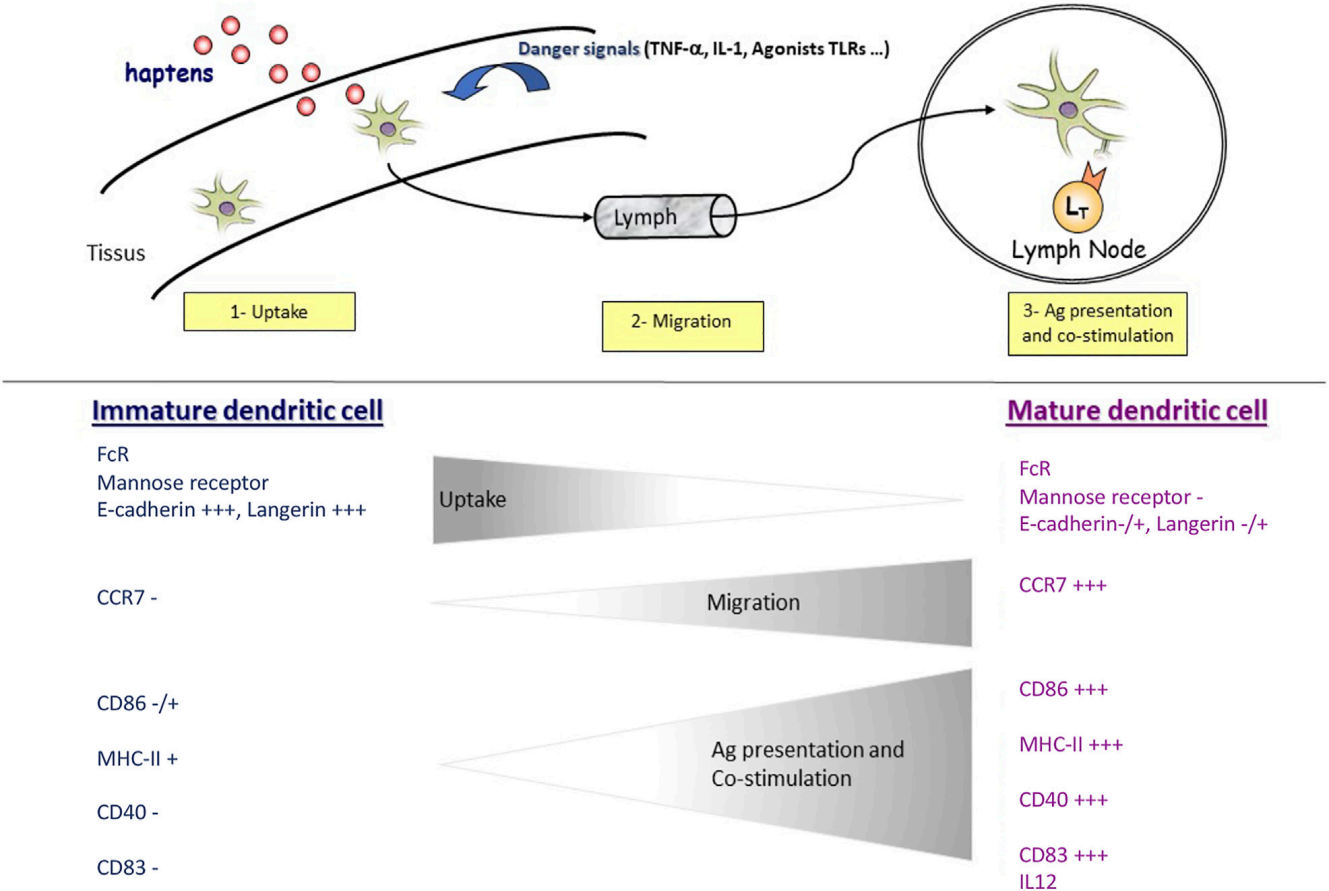
In response to haptens and/or danger signals, immature DCs are activated into a mature phenotype. Immature DCs can capture and reprocess antigens. When DCs are activated, they lose this function and acquire the ability to migrate upon CCR7 expression. During this same activation, DCs also express specific clusters of differentiation (CDs) involved in lymphocyte co-stimulation such as CD86 and interaction with T cells such as CD83. In addition, DCs can produce cytokines such as IL-12 and IL-23, participating in LT polarization.

DCs are divided into conventional DCs (cDC) and plasmacytoid DCs (pDC). Healthy skin contains mainly LCs in the epidermis and cDC in the dermis, and very few pDC (Collin and Bigley, 2018). Moreover, DCs exist in two phenotypically and functionally distinct states: immature DCs and mature DCs. Immature DCs have high phagocytosis activity and are specialized in Ag uptake and processing. In contrast, mature DCs cannot phagocyte Ags but are dedicated to stimulating Ag-specific naive T cells in the LN. In response to an Ag, activated DCs express the CCR7 receptor, which allows their migration to the draining LN *via* a gradient of chemokines such as CCL19 and CCL21 (Ohl et al., 2004; Lian and Luster, 2015). These two distinct states of the DC phenotype during the process of activation by an antigen or a hapten (Figure 1) allow studying their activation *in vitro* by monitoring extracellular markers.

Due to their rarity in tissues, much of the biology of DCs has been determined from studies of cells grown *in vitro* from hematopoietic precursors using growth factors (Helft et al., 2015). Recent studies have thus established numerous methods for generating different types of DCs by culturing mouse bone marrow (BM) cells, human cord blood precursors, or peripheral blood monocytes using specific cytokines to control and shape their differentiation (Ohta et al., 2008; Inaba et al., 2009; Sichien et al., 2017). In this context, three different models of DCs will be addressed: 1) CD34-DCs derived from CD34^+^ hematopoietic progenitor cells (Caux et al., 1997), 2) Mo-DCs derived from blood monocytes (Geissmann et al., 2008), and 3) BM-DCs derived from mouse bone-marrow (Domínguez and Ardavín, 2010).

The use of DCs models responds to different issues. It helps to limit animal testing and responds to ethical and regulatory pressures to reduce or prohibit the use of animal models for safety testing. This has played a key role in the development of alternative methods using DCs models. This review will therefore focus on protocols for the generation of DCs in the context of skin sensitization assessment.

## Human Dendritic Cells Derived From CD34^+^ Hematopoietic Progenitor Cells (CD34-DC)

### Materials and Equipment

Citrate Na Cord Blood.

Phosphate-Buffered Saline (PBS), pH 7.4 (Gibco, Thermo Fisher Scientific, Cat. No. 10010056).

Ficoll-Paque PLUS density gradient media (GE Healthcare, Cat. No. 17-1440-03).

Heat-inactivated (30 min, 56°C) fetal calf serum (FCS).

RPMI 1640 (Gibco, Thermo Fisher Scientific, Cat. No. 72400-021).

CD34 MicroBead kit (Miltenyi Biotec, Cat. No. 130-046-702).

Nickel (II) sulfate hexahydrate (NiSO_4_ 6H_2_O) (Sigma Aldrich, Cat. No. N4882).

Complete RPMI medium: RPMI 1640 supplemented with 10% of inactivated FCS.

MACS-Buffer: PBS-0.5% EDTA (2 mM), 0.05% FCS.

Human recombinant Flt3-Ligand (Miltenyi Biotec, Cat. No. 130-096-474).

Human recombinant GM-CSF (Miltenyi Biotec, Cat. No. 130-093-868).

Human recombinant TNF-α (Miltenyi Biotec, Cat. No.130-094-014).

Human recombinant IL-4 (Miltenyi Biotec, Cat. No. 130-093-924).

Human recombinant TGF-β (Miltenyi Biotec, Cat. No. 130-095-067).

Human recombinant SCF (Miltenyi Biotec, Cat No. 130-096-693).

APC mouse anti-human CD86 (BD Biosciences, Cat. No. 555660).

PE mouse anti-human CD83 (BD Biosciences, Cat. No. 556855).

PE mouse anti-human CD197 (CCR7) (BD Biosciences, Cat. No. 560765).

PE mouse anti-human CD207 (Langerin) (Beckman Coulter, Cat. No. IM3577).

FITC mouse anti-E-Cadherin (BD Biosciences, Cat. No. 612130).

15 and 50 ml polypropylene conical tubes.

Beckman centrifuge.

## Methods

### Isolation Mononuclearcells By Ficoll-Paque Gradient Centrifugation

All operations will be carried out under sterile conditions, using only sterile media, instruments, pipette tips, and culture dishes.

1) Place fresh citrate-Na cord blood into tissue culture flask. Using a sterile pipet, add twice the volume of room-temperature PBS. Mix well. Put 15 ml of Ficoll-Paque solution in 50 ml conical centrifuge tubes.
2) Add slowly 25 ml of blood/PBS mixture by placing the tip onto the top of the Ficoll-Paque. Important: When stratifying the sample, do not mix the Ficoll-Paque medium solution and the diluted blood sample.
3) Centrifuge 30 min at 800g, 18°C-20°C, without brake.
4) Using a sterile pipet, remove the upper layer containing the plasma and most of the platelets. Using another pipet, transfer the mononuclear cell layer to another centrifuge tube. Wash cells by adding PBS-2%FCS (3 times the volume of the mononuclear cell layer) and centrifuging 10 min at 300 g, 18–20°C with a low brake. Discard the supernatant, resuspend cells in PBS-2%FCS, and repeat the wash once to remove most of the platelets with brake.

The washing steps described above remove most of the platelets from the mononuclear cell suspension. When platelets are very abundant, additional steps are needed to remove the extra platelets in the peripheral blood. Add 3 ml of FCS to a centrifuge tube for each milliliter of mononuclear cells. Layer the cell suspension (1-2 x 10^7^cells/ml) over the FCS. Centrifuge 15 min at 200 g, 18°C to 20 °C. discard the supernatant containing the platelets. Resuspend cell pellet in complete RPMI-10 and proceed as in step 5.

5) Resuspend mononuclear cells in a complete RPMI medium. Count cells and determine viability by trypan blue exclusion.

If desired, determine the purity of the PBMC population by flow cytometry.

### Isolation of CD34 Positive Cells

6) Wash cells once with MACS buffer at 300 g for 10 min. Resuspend cell pellet in a final volume of 300 µl of MACS buffer for up to 10^8^ total cells.
7) Add 50 µl of FcR blocking Reagent for up to 10^8^ total cells.
8) Add 50 µl of CD34 Micro Beads for up to 10^8^ total cells.
9) Mix well and refrigerate for 30 min (4°C–8°C) with very gentle agitation.
10) Wash cells by adding 10 ml of MACS buffer and centrifuge at 350 g for 10 min, at 4°C. Aspirate the supernatant completely. Wash twice.
11) Resuspend up to 10^8^ total cells in 500 ml of MACS buffer.

### Magnetic Separation With MS or LS Columns

12) Place column in the magnetic field of a suitable MACS separator:

MS: 2.10^8^ total cells LS: 2.10^9^ whole cells.

13) Prepare column by rinsing with the appropriate amount of MACS-buffer:

MS: 500 µl LS: 3 ml

14) Apply cell suspension onto the column.
15) Once the unlabeled cells have passed through the column, wash the column with the appropriate buffer. To do this, add buffer only when the column reservoir is empty and perform the washing steps by adding buffer three times.

MS: 3 × 500 µl LS: 3 x 3 ml

16) Remove the column from the separator and place it on a new suitable collection tube.
17) Pipette an appropriate amount of buffer onto the column. Immediately flush out the magnetically labeled cells by firmly pushing the plunger into the column:

MS: 2 × 500 µl LS: 2 x 2.5 ml

The purity of the isolated hematopoietic progenitor cells can be evaluated by flow cytometry. CD34^+^ cells can be analyzed by direct immunofluorescent staining using an antibody recognizing an epitope different from that recognized by the CD34 monoclonal antibody MicroBead kit.

### Generation of Dendritic Cells From CD34^+^ Cells

18) Resuspend the CD34^+^ cells to a density of 150.10^3^ cells per ml in medium containing 200 U/ml GM-CSF, 50 ng/ml SCF, 50 U/ml TNF-α and 50 ng/ml de Flt3-L.

The SCF is an option to increase the number of cells, but it is not mandatory.

Incubate the cells in 12 wells plate if less than 150.10^3^ cells or flask culture if more than 150.10^3^ cells.

19) After 4 days, the volume of the medium is doubled to allow cells expansion by adding media supplemented with 200 U/ml GM-CSF and 50 U/ml TNF-α.
20) From day 5, dilute cells in a double volume every day without adding any cytokines.
21) On day 7, count cells and determine viability by trypan blue exclusion.

The phenotype of dendritic cells derived from CD34^+^ (CD34^+^-DC) can be evaluated by flow cytometry with immunofluorescent staining using antibodies recognizing CD1a, CD86, HLA-DR, and CD14. At day 7, depending on the donor, our validation criteria are the following: <2.5% of the cells were CD34^+^, 40–50% were CD1a+, 15–20% were CD14^+^, 50–60% were CD86 + low, 90–95% were CD40 + low, 90–95% were HLA-DR + low, <5% were CD83^+^, and <2.5% were CCR7+.

### Generation of Langerhans Cells Like From Human CD34^+^ Hematopoietic Progenitors

1) Resuspend the CD34^+^ cells to a density of 150.10^3^ cells/ml in media containing 200 U/ml GM-CSF, 50 ng/ml SCF, 50 U/ml TNF-α, 50 ng/ml Flt3-L and 10 ng/ml TGF-β.
2) Incubate cells in 12 wells plate if less than 150.10^3^ cells or flask culture if more than 150.10^3^ cells.
3) Four days later, cells are expanded in two volumes. The volume added contains 200 U/ml GM-CSF and 50 U/ml TNF-α.
4) On day 5, dilute cells in a double volume every day with 10 ng/ml TGF-β.
5) On day 7, count cells and determine viability by trypan blue exclusion.

The phenotype of dendritic cells derived from CD34^+^ (CD34^+^-LC like) can be evaluated by flow cytometry with immunofluorescent staining using antibodies recognizing CD1a, CD86, E-cadherin, HLA-DR, CD207 (specific marker for Langerhans cells), and CD14.

### Chemical Treatment of Immature DC

On day 7, DC were washed three times before treatment with NiSO_4_ (500 μM, Sigma, St Louis, MO) for 24 h.

### Flow Cytometry Analysis

Cultured DC were re-suspended at 2.5 × 10^5^ cells in 30 μl of culture medium and incubated for 30 min at 4°C with monoclonal antibodies (mAbs) or appropriate isotypic controls. After three washes in cold phosphate-buffered saline (PBS) supplemented with 0.5% of BSA, cells were fixed with 1% paraformaldheyde in PBS. The following mAbs were used: anti-E-cadherin (HECD-1, R&D systems), anti-Langerin (DCGM4, Immunotech), anti-CD86 (2331, BD Biosciences), anti-CCR7 (2H4, BD Biosciences) and PE-conjugated anti-CD83 (HB15a, Immunotech).

A second step including a goat anti-mouse IgG-FITC (0819, Immunotech) was added fo anti-E-cadherin, anti-Langerin and anti-CD86. In the case of anti-CCR7, a goat anti-mouse IgM (Alexa Fluor 633, Molecular Probes, Leiden, Netherlands) was used for the second step.

All antibodies were diluted at 1:100, except for CD86, which was used at a 1:20 dilution. Forward and side scatter analysis (FSC vs. SSC) was performed to exclude debris, followed by side scatter height (SSC-H) vs. side scatter area (SSC-A) analysis to focus on singlets. Next, one-parameter histograms to identify cells with a particular marker expression or two-parameter density plots for further analysis were performed.

Appropriate isotypes controls were used at the same concentration as the test antibody to determinate the positive cells.

For each sample 10^4^ cells were analyzed. Cell fluorescence was acquired using the Attune Nxt cytometer (Thermo Fisher Scientific), and further analyzed with FlowJo software (Becton Dickinson).

### Results

**FIGURE 2:**
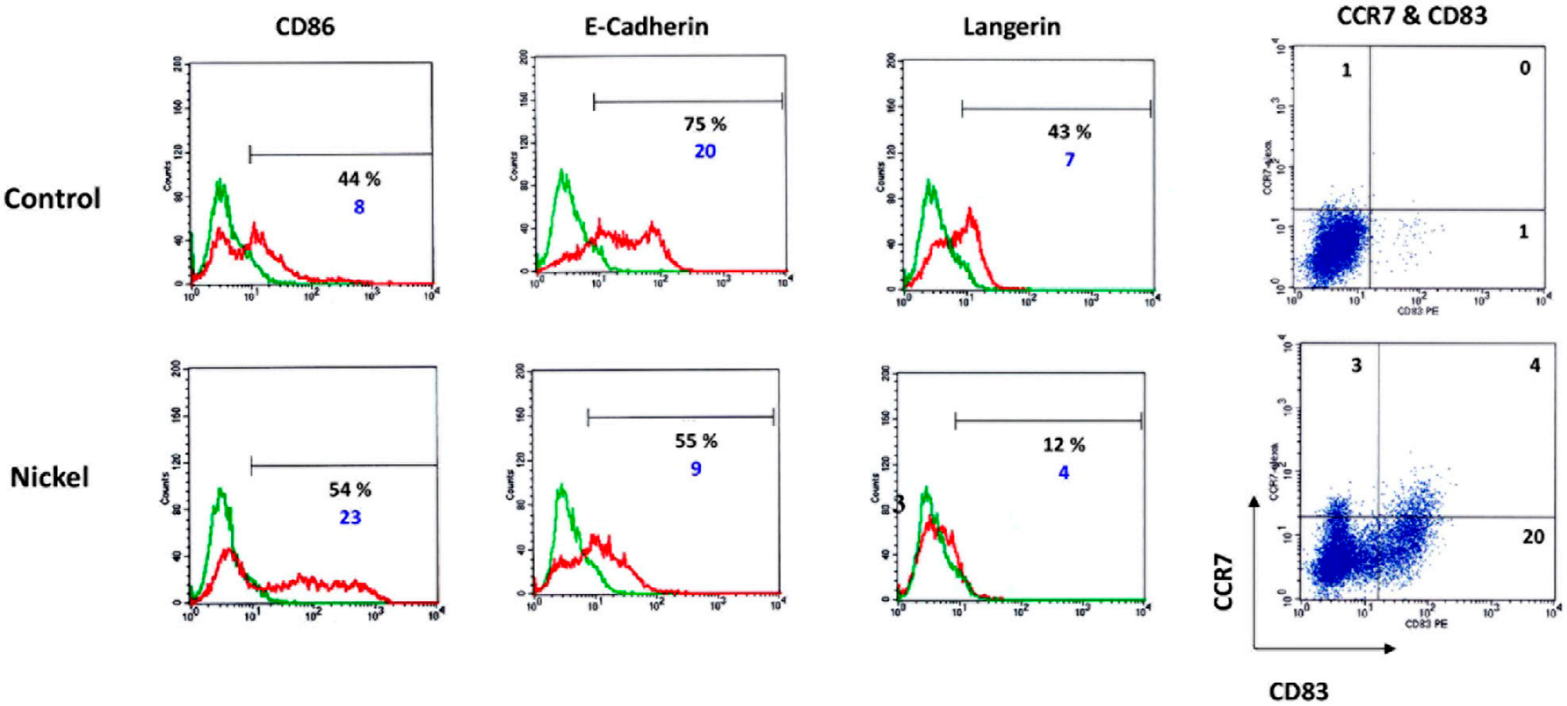
DCs were generated by culturing CD34^+^ HPC from cord blood in the presence of GM-CSF, TNF-α, and Flt-3L for 7 days. Cells were then washed and stimulated or not (control) by NiSO4 (500 μM) for 24 h before being analyzed by flow cytometry for the expression of CD86, E-Cadherin, Langerin. Numbers represent the % of positive cells (black number) and cMFI (blue number), which is the ratio between the total MFI obtained using the specific antibody (green lines) to the total MFI obtained using a control fluorescent antibody of the same isotype (red lines). For CD83 and CCR7, two-dimensional plots show the surface expression profile of DC for CD83 and CCR7 or control antibodies. Quadrant position was determined using control Abs for each condition of stimulation. Numbers in each quadrant represent cell percentages.

Immature DCs were generated from CD34^+^ cells. Hematopoietic progenitor cells (HPC) cultured in the presence of granulocyte-macrophage-colony stimulating factor (GM-CSF), TNF-α, and Flt-3L for 7 days. As shown by flow cytometry studies, a significant proportion of these cells express the specific markers of LC: CD1a (70%), E-cadherin (75%), and Langerin/CD207 (43%) known to be associated with the Birbeck granules. On day 7, these cells are in an immature state, as shown by the low expression of CD83 (1% of positive cells). When CD34-DCs are treated with 500 mM of NiSO_4_ for 24 h, extensive phenotypic changes occur with the upregulation of CD86 and CD83 and the downregulation of E-cadherin and Langerin (Figure 2). The viability of the cells after NiSO_4_ treatment was not inferior to 80% as assessed by trypan blue exclusion (data not shown).

## Human Dendritic Cells Derived Monocytes (Mo-DC)

### Materials and Equipment

Ficoll-Paque PLUS density gradient media (GE Healthcare, cat. no. 17144003).

Phosphate-Buffered Saline (PBS) supplemented with 0.5% FCS and 2 mM EDTA.

CD14 microbeads (Miltenyi Biotech).

RPMI 1640 (Gibco, Thermo Fisher Scientific, cat. no. 72400-021).

Heat-inactivated fetal calf serum (FCS) (30 min, 56°C).

Pen strep (Gibco, Thermo Fisher Scientific, Cat. No. 15140122).

Sodium pyruvate (NaPy) (Gibco, Thermo Fisher Scientific, Cat. No. 11360070).

Lipopolysaccharide (LPS) (Sigma-Aldrich, Cat. No. L6529).

Complete RPMI medium: 500 ml of RPMI-1640 supplemented with 50 ml of heat-inactivated FCS, 1 mM NaPy, 100 U/ml of penicillin, and 100 μg/ml streptomycin sulfate (P/S).

Recombinant human-Granulocyte-Macrophage Colony-Stimulating Factor (rh-GMCSF) (Miltenyi Biotec 130-093-868).

Recombinant human Interleukine 4 (rhIL-4) (Miltenyi Biotec 130-093-924).

APC mouse anti-human CD86 (BD Biosciences, Cat. No. 555660).

PE mouse anti-human CD83 (BD Biosciences, Cat. No. 556855).

Beckman centrifuge.

50 ml Leucosep tubes (Dutscher Blood September 016,780).

LS columns and magnetic kit (Miltenyi Biotec, Cat. No. 130-042-401).

## Methods

### Generation of DCs From Human Monocytes (Mo-DCs)

All operations will be carried out under sterile conditions, using only sterile media, instruments, pipette tips, and culture dishes.

#### Isolation of Mononuclear Cells by Ficoll-Paque Gradient Centrifugation

1) Place 15 ml of Ficoll-Paque solution in leuco-September 50 ml conical centrifuge tubes.
2) Add slowly 30 ml of fresh blood by placing the tip of the pipet up of the Ficoll/Paque, inclining it.
3) Centrifuge 10 min at 800 g, 18°C-20°C, without brake.
4) Using a sterile pipet, remove the upper layer containing the plasma and most of the platelets. Transfer the mononuclear cell layer to another 50 ml centrifuge tube using another pipet. Wash cells by adding PBS up to 50 ml centrifuging 10 min at 300 g, 18–20°C with brake. Remove the supernatant, resuspend cells in PBS, and repeat the wash.
5) Resuspend mononuclear cells in complete RPM 1640. Count cells and determine viability by trypan blue exclusion.

If desired, determine the purity of the PBMC population by flow cytometry.

At this step, if too many platelets are present, complete with PBS and centrifuge 15 min at 200 g, 18°C to 20 °C, discard the carefully supernatant containing the platelets by aspiration. Resuspend cell pellet in complete RPMI-10 and proceed as in step 6.

#### Isolation of CD14 Positive Cells

6) Wash cells once with MACS buffer at 300 g for 10 min. Resuspend cell pellet in a final volume of 800 µl of MACS buffer for up to 10^8^ total cells.
7) Add 80 µl of CD14 Micro Beads for up to 10^8^ total cells.
8) Mix well and refrigerate for 15 min (4°C–8°C).
9) Wash cells by adding 10 ml of MACS buffer and centrifuge at 350 g for 10 min, at 4°C. Aspirate the supernatant completely. Wash twice.
10) Resuspend up to 10^8^ total cells in 500 µl of MACS-buffer

#### Magnetic Separation With is Columns

11) Place LS column (maximum 2.10^9^ total cells) in the magnetic field of a suitable MACS separator.
12) Prepare column by adding 3 ml of MACS-buffer.
13) Apply cell suspension into the filter of the top of the column.
14) Collect unlabeled cells that pass through
15) Wash the column three times with 3 ml of MACS buffer. Wait until the column is empty before repeating the operation by adding a fresh buffer.
16) Collect total effluent; this is the unlabeled cell fraction.
17) Remove the column from the separator and place it on a suitable collection tube.
18) Pipette 2 × 5 ml of buffer onto the column. Immediately flush out the magnetically labeled cells by firmly pushing the plunger into the column.

The purity of the isolated monocytes cells can be evaluated by flow cytometry. CD14 ^+^ cells can be analyzed by direct immunofluorescent staining using an antibody recognizing an epitope different from that recognized by the CD14 monoclonal antibody MicroBead.

#### Generation of Dendritic Cells From CD14^+^ Cells

19) Resuspend the CD14^+^ cells to a density of 1.10^6^ cells/ml in fresh media containing 550 U/ml GM-CSF and 550 U/ml of IL-4.

Incubate the cells in T175 flasks at one million per? milliliter.

20) Four days later, count cells and determine viability by trypan blue exclusion.

The phenotype of dendritic cells derived from CD14^+^ can be evaluated by flow cytometry with immunofluorescent staining using antibodies recognizing CD1a, DC-SIGN, CD86, and CD83. On days 4-5, depending on the donor, our criteria are the following: 50–100% were CD1a+, 90–100% were DC-SIGN+, CD86, and CD83 expression were below 30% and 5%, respectively.

### Chemical Treatment of Immature DCs

Mo-DCs (at day 5) were washed three times in RPMIc, and their concentration was adjusted to 1 × 10^6^ cells/ml. iDCs were stimulated or not with NiSO_4_ (500 μM, Sigma, St Louis, MO) or with DNCB (5 mM, Sigma, St Louis, MO) for 24 h.

### Flow Cytometry Analysis

Cultured DC were re-suspended at 2 × 10^5^ cells in 100 μl of PBS-2%FBS and incubated for 25 min at 4°C with monoclonal antibodies (mAbs) or appropriate isotypic controls. After three washes in cold phosphate-buffered saline (PBS). The following mAbs were used: anti-CD86 (2331, BD Biosciences) and PE-conjugated anti-CD83 (HB15a, Immunotech).

All antibodies were diluted at 1:100, except for CD86, which was used at a 1:20 dilution. Forward and side scatter analysis (FSC vs. SSC) was performed to exclude debris, followed by side scatter height (SSC-H) vs. side scatter area (SSC-A) analysis to focus on singlets. Next, one-parameter histograms to identify cells with a particular marker expression or two-parameter density plots for further analysis were performed.

Appropriate isotypes controls were used at the same concentration as the test antibody to determinate the positive cells. For each sample 10^4^ cells were analyzed. Cell fluorescence was acquired using the Attune Nxt cytometer (Thermo Fisher Scientific), and further analyzed with FlowJo software (Becton Dickinson).

### Results

**FIGURE 3:**
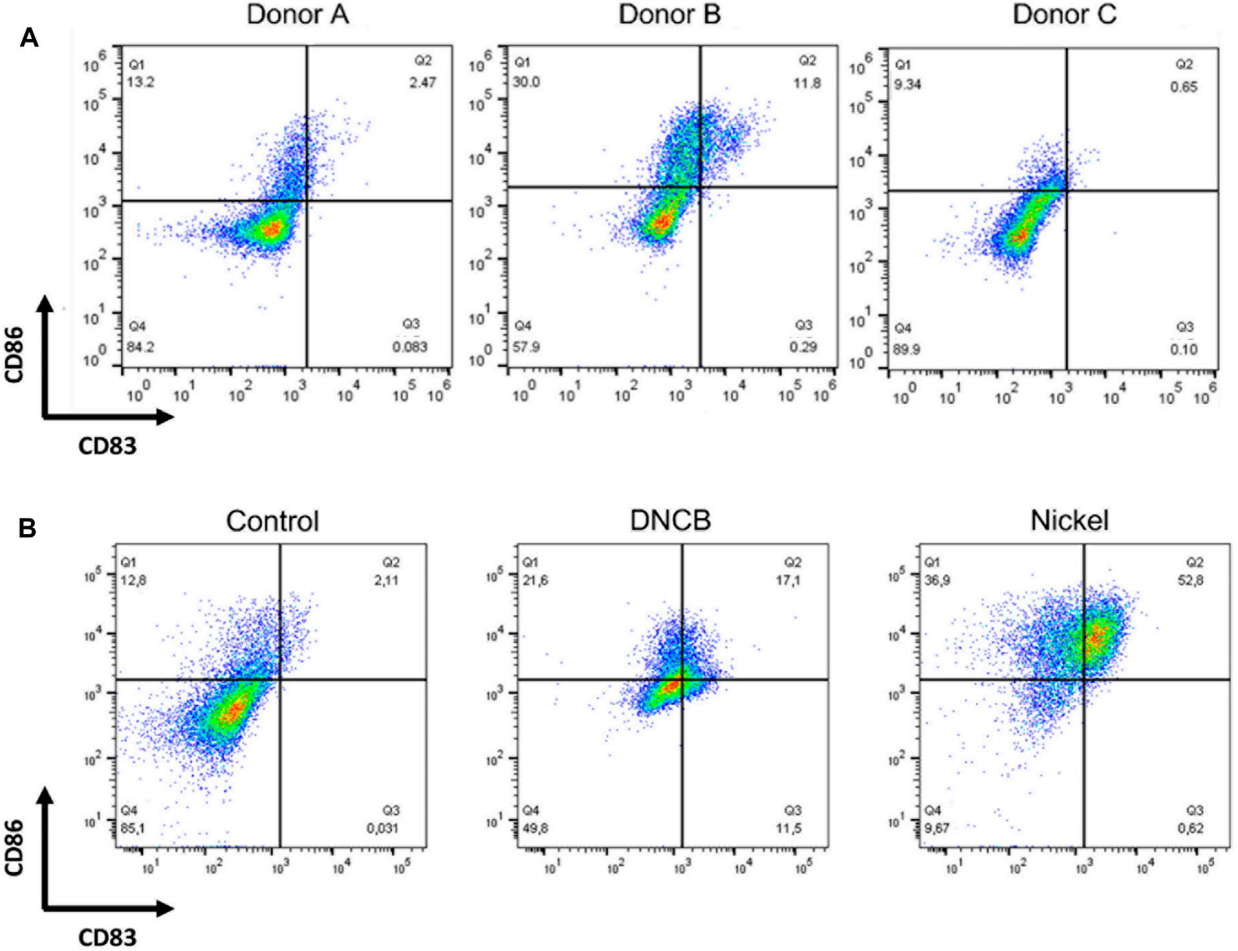
DCs were generated by culturing CD14^+^ from human blood donors in the presence of GM-CSF, and IL-4 for 4 days **(A)** Cells were then counted, washed, and analyzed by flow cytometry at steady-state for the expression of the following cell surface markers: CD86 and CD83 **(B)** Cells were then washed and stimulated or not (control) by DNCB (5 µM) or NiSO_4_ (500 μM) for 24 h before being analyzed by flow cytometry for the expression of the following cell surface markers: CD86 and CD83. Quadrant position was determined using isotype antibodies for each condition of stimulation. Numbers in each quadrant represent cell percentages.

Immature DCs were generated from CD14 ^+^ cells cultured in the presence of GM-CSF and IL-4 for 4 days. On day 4, depending on the donor, more than 90% of the cells were DC-SIGN positive; 40–90% expressed CD1a depending on the donor, while less than 5% of the cells were CD83 positive and 20–30% were CD86 positive depending on the donor. As shown in Figure 3A, depending on the donor, Mo-DCs often present a great heterogeneity regarding basal CD86 expression. CS promote Mo-DCs maturation as shown by the increased expression of CD86 and CD83 markers after 24 h of exposure to DNCB (2,4-dinitrochlorobenzene) (5 µM) or NiSO_4_ (500 µM) compared with control cells (Figure 3B).

## Murine Dendritic Cells Derived Mouse Bone Marrow Progenitors (BM-DC)

### Materials and Equipment

Eight- to 14-week-old C57BL/6J mice.

PBS, pH 7.4 (Gibco, Thermo Fisher Scientific, Cat. No. 10010056).

IMDM culture medium (Gibco, Thermo Fisher Scientific, Cat. No. 12440053)*

Heat-inactivated Fetal Calf Serum (56°C, 30 min).

Penicillin/streptomycin (Gibco, Thermo Fisher Scientific, Cat. No. 15140122).

Recombinant mouse GM-CSF (Miltenyi Biotec, Cat. No. 130-095-793) or conditioned medium from genetically engineered GM-CSF expressing J558 cells

β-mercapto-ethanol (Gibco, Thermo Fisher Scientific, Cat. No 31350010).

Red blood cell lysis buffer.

*Of note: *RPMI 1640* (Gibco, Thermo Fisher Scientific, cat. no. 72400-021) *could also be used to differentiate bone marrow progenitors in BM-DCs.*

Lipopolysaccharide (LPS) (Sigma-Aldrich, Cat. No. L6529).

Complete IMDM medium: IMDM free medium supplemented with 10% of inactivated FCS, 100 U.ml^−1^ penicillin, and 100 μg ml^−1^ streptomycin sulfate.

Differentiation IMDM medium: Complete IMDM medium supplemented with 20 ng ml^−1^ recombinant murine GM-CSF and 25 µM β-mercaptoethanol.

Red blood cell lysis buffer recipe: dissolve in 1 L of ultra-pure H_2_O, 8.32g ammonium chloride (NH_4_Cl), 0.82g sodium bicarbonate (NaHCO_3_), and 0.043g EDTA. Adjust pH to 7.2–7.4. Mix well and filter. Autoclave the solution. Store up to 6 months at 4°C.

FITC anti-mouse CD86 (BD Pharmingen, Cat. No. 553691).

PE-Vio770 anti-mouse MHC-II (Miltenyi Biotec, Cat. No. 130-112-232).

APC anti-mouse CD11c (Miltenyi Biotec, Cat. No. 130-110-702).

100 mm Petri dish.

15 ml and 50 ml polypropylene conical tubes.

5 ml syringe.

26-gauge needles.

70 μM cell strainer.

Beckman centrifuge.

## Methods

### Isolation of Bone Marrow Progenitor Cells for Differentiation in BM-DCs

#### Hind Limbs Recovery

1) According to the project, choose a mouse with the desired genetic background and the expected genetic modifications.
2) Mice must be handled and sacrificed in accordance with the principles and procedures outlined in Council Directive.
3) Once sacrificed, place the mouse on its back with its forelimbs and its hind legs pinned into a dissection tray.
4) Clean the peeling with 70% ethanol before operating the dissection.
5) Cut the skin at the base of the abdomen and then make a diagonal cut alongside each of the hindlimbs to access tissues and bones.
6) Clean off as much muscle as possible using small dissection scissors to free the leg bones and expose the femur and tibia.
7) Transfer the legs into a 50 ml polypropylene conical tubes containing cold PBS.

From this stage on, all operations will be carried out under sterile conditions, using only sterile media, instruments, pipette tips, and culture dishes.

#### Samples Sterilization

8) Under the microbiological safety cabinet, transfer bones from the PBS to a small culture dish or tube filled with 70% cold ethanol for 10 s.
9) Remove samples from ethanol and then perform three successive washes with cold PBS.
10) Transfer bones into a 100 mm Petri dish containing 2 ml of serum-free IMDM culture medium.
11) Cut the knee joints with dissection scissors to separate each femur and tibia.
12) Then, carefully grasp the different osseous segments with dissection tweezers and carefully cut the epiphyses (ends of the bones) to access bright red bone marrow contained in the center of the bones.
13) Once processed, transfer each bone in a clean 100 mm Petri dish containing 10 ml of serum-free IMDM-medium.

#### Bone Marrow Recovery

14) Mount a 26-gauge needle on a 5 ml sterile syringe previously filled with medium from the dish containing the samples.
15) Hold the bone with sterile tweezers above the dish of sterile media and insert the needle into one side of the bone to flush bone marrow cells into the 100 mm Petri dish.
16) The bone marrow washes out, either in small pieces or as a single piece. It should be flushed out of the bone and into the dish of sterile media.
17) Repeat this step as needed to completely wash the marrow out of the bone. When the bone is clean, it will be white and translucent.
18) Repeat this procedure with the remaining bones. Once cleaned, discard each empty bone.
19) Place a 70 μm cell strainer at the top of a 50 ml tube and proceed to the transfer of the cells to filter remaining particles such as hairs and debris.

If bone marrow is still intact, carefully pipette media up and down into the dish to obtain a single cell suspension before the transfer.

20) Centrifuge cells at 300 g for 10 min. Remove and discard supernatant carefully.

#### Red Blood Cell Lysis (Optional)

21) Resuspend cell pellet in 2 ml of red blood cells lysis buffer and incubate for exactly 2 min.
22) Complete to 50 ml with serum-free IMDM medium.
23) Centrifuge cells at 300 g for 10 min.
24) Remove and discard the supernatant.
25) Resuspend the progenitor cells in 20 ml of complete IMDM medium.

#### *In Vitro* Differentiation of Dendritic Cells

26) Count viable cells after trypan blue staining.
27) Plate the cells at a density of 3.10^6^ viable cells into 10 ml of IMDM differentiation medium per 100 mm Petri dish.
28) Place dishes in a 37°C incubator with 5% CO_2_.

Regarding the use of conditioned medium from genetically engineered J558 cells expressing GM-CSF, an ELISA test must be performed to determine the concentration of GM-CSF for each batch of J558 supernatant. Optimal concentration could also be determined empirically by performing a serial dilution of the J558 conditioned medium and monitoring phenotype, viability, and maturation.

#### Culture Care and Maturation

29) On day 3, add 10 ml of IMDM differentiation medium supplemented with 20 ng/ml (700–1000 U/ml) GM-CSF and 25 µM β-mercaptoethanol to reach a final volume of 20 ml per dish.
30) On day 7, refresh half of the media. Take 10 ml in each Petri dish. Centrifuge at 300 g for 10 min at room temperature. Resuspend cells in fresh IMDM differentiation medium and dispense 10 ml in each plate.

The phenotype of DCs derived from bone marrow progenitor cells can be evaluated by flow cytometry with immunofluorescent staining using antibodies recognizing CD11b, CD11c, MHC class II, F4/80, CD83, and CD86. At day 8-9, our validation criteria for BM-DC differentiation are the following: over 90% viable cells, over 70% cells expressing CD11c, and less than 20% CD86 positive cells.

### Chemical Treatment of BM-DC

BM-DC were washed twice and then incubated at 1 × 10^6^ cells/ml. Cells were then treated with 100 µM Cinnamaldehyde (CinA) (Sigma-Aldrich, cat. No. W228613), or 25 ng/ml lipopolysaccharide (LPS) (Sigma-Aldrich, cat. No. L2630) as a positive control of BM-DC activation for 24 h. CinA was dissolved in DMSO at 0.1% as a final concentration in complete medium.

### Flow Cytometry Analysis

For each condition, cultured DC were washed and re-suspended at 4 × 10^5^ cells in 100 μl of PBS supplemented with 0.5% BSA and 2.5 mM EDTA and incubated for 25 min at 4°C with monoclonal antibodies (mAbs) or appropriate isotypic controls. After three washes in cold phosphate-buffered saline (PBS) supplemented with 0.5% of BSA and 2.5 mM EDTA, the following mAbs were used: anti-MHC-II (Miltenyi Biotec), anti-CD11c (Miltenyi Biotec) and anti-CD86 (BD Pharmingen). All antibodies were diluted at 1:100, except for CD86, which was used at a 1:20 dilution. Forward and side scatter analysis (FSC vs. SSC) was performed to exclude debris, followed by side scatter height (SSC-H) vs. side scatter area (SSC-A) analysis to focus on singlets. Next, one-parameter histograms to identify cells with a particular marker expression or two-parameter density plots for further analysis were performed.

Appropriate isotypes controls were used at the same concentration as the test antibody to determinate the positive cells. For each sample, 10^4^ cells were analyzed. Cell fluorescence was acquired using the Attune Nxt cytometer (Thermo Fisher Scientific), and further analyzed with FlowJo software.

### Results

**FIGURE 4:**
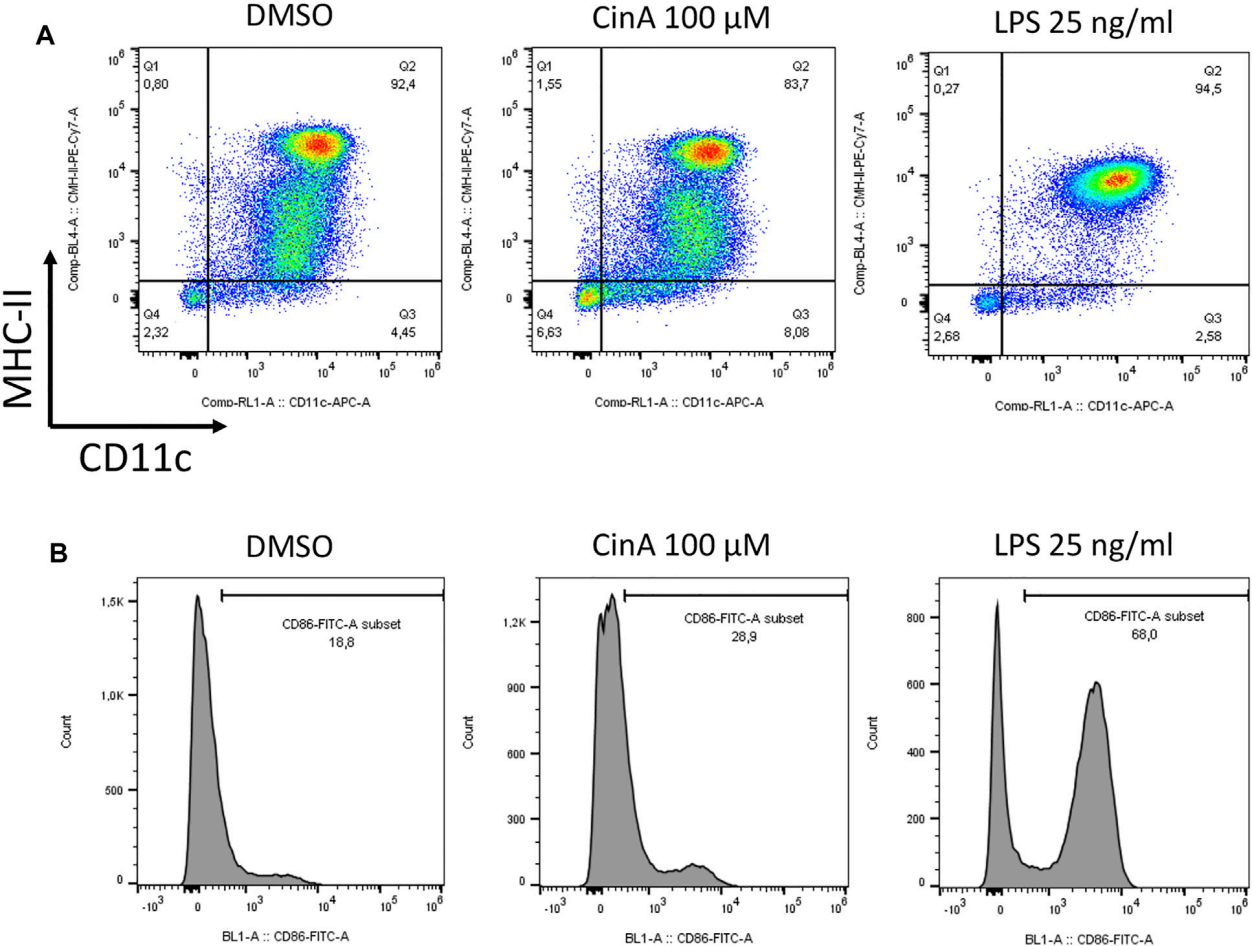
BM-DCs were generated by culturing mouse progenitor cells in the presence of GM-CSF for seven to 8 days. Cells were then washed and stimulated or not (control) for 24 h with CinA (100 µM), or LPS (25 ng ml^−1^) as a positive control of DC activation before being analyzed by flow cytometry for the expression of the following cell surface markers **(A)** CD11c and MHC-II, or **(B)** and CD86. Quadrant position and histogram gates were determined using isotype antibodies for each stimulation condition. Numbers in each quadrant/histogram gate represent cell percentages.

Mouse bone-marrow progenitor cells cultured with GM-CSF differentiate in BM-DCs. A significant proportion of these cells express the following DCs cell surface markers: CD11c, CD11b, and MHC-II, while they show a relatively low expression level for the macrophage marker F4/80. In immature DCs, MHC-II, CD83, and CD86 are expressed at low levels on the cell surface. At a steady-state, the BM-DCs population shows different levels of MHC-II expression. However, as shown by flow cytometry studies (Figure 4), BM-DCs treated with LPS at 25 ng/ml for 24 h as a positive control for DC maturation show significant phenotypic changes with a shift towards high MHC-II expression as well as a significant increase in CD86 expression. Exposition to CS also induces BM-DCs maturation, as shown by the increase of CD86 expression after 24 h of exposure to 100 µM CinA.

## Discussion

DCs are specialized sentinels responsible for coordinating innate and adaptative immunity. Immature and mature DCs display great morphological, phenotypical, and functional differences that can be followed (Hargadon, 2016). Monitoring DCs activation *in vitro* is mainly based on studying cell surface markers using flow cytometry. Cytokines’ expression and secretion, capacity for internalization by phagocytosis, or even their ability to activate lymphocytes in co-culture could provide useful tools to study DCs’ cellular response toward CS. These modifications help to study DCs activation upon CS exposure.

Numerous studies have been conducted with different models of DCs to study their activation by CS. Various alternative methods have been developed to this extent for the detection of contact allergens (Mehling et al., 2012).

Our review provides different protocols for the generation of human and murine DCs. DCs could be obtained *in vitro* by deriving DCs from progenitors or monocytes in humans or mice. With these technical approaches, a relatively large number of DCs can be generated, allowing to decipher the underlying mechanistic events of DCs activation. However, each of these biological models has its own specificities, which we will briefly discuss in the context of skin sensitization.

In the CD34-DC model, regardless of the donor that allowed differentiation, there is little variability in the response (Boislève et al., 2005; Larangé et al., 2009). DCs respond to CS with comparable inductions. This model is thus reproducible and less expensive and can provide numerous cells to perform experiments. However, access to blood from umbilical cord blood is restricted depending on the country of origin.

In addition, the CD34-DC model allows the differentiation of DCs mimicking dermal DCs and LCs that are epidermal DCs with the same batch of progenitors. The activation of the two DC populations by the same CS can then be studied.

Regarding the Mo-DCs model, numerous studies have been conducted on the effects of CS on Mo-DCs (Aiba et al., 1997; Antonios et al., 2010). CS can induce phenotypic modifications of immature Mo-DCs. Nickel and DNCB (2,4-dinitrochlorobenzene) can significantly upregulate the surface expression of the markers CD54, CD86, and HLA-DR. However, a significant limitation in this model is the considerable variation from donor to donor. For illustration, CD86, a marker of DCs activation induced by CS, is often highly expressed on immature DCs. CD86 expression exceeding 30% at steady state on immature DCs results in excluding some donors on the base of this endpoint due to inappropriate activation. In this context, basal CD86 expression should be included to compare the response to a CS between different donors.

The model of DC-derived from mouse bone marrow progenitors (BM-DCs) allows to generate a huge amount of immature DCs. Due to the large amount of DCs that can be generated from a single animal, the BM-DCs model meets at least one of the criteria of the 3Rs and is therefore considered an alternative method. Moreover, BM-DCs are derived from congenic mice; therefore, inter-donor variability is limited, and a better reproducibility is obtained, which is a great advantage compared to human DCs. A limitation of this model is its inability to detect the CS triggering their effects *via* human TLR4. Indeed, mice and humans show differences in recognition *via* TLR4 for metals such as Nickel (Schmidt et al., 2010).

## Conclusion

Skin sensitization is a highly complex and dynamic process. All three methods to obtain differentiated human or murine DCs are suitable for evaluating the effects of CS in the context of KE3. However, depending on the source of the DCs, some CS that are signaling *via* TLRs may not be correctly identified.

Unfortunately, the production of DCs isolated from progenitors or monocytes is expensive. Because of the inherent variability between human donors, the need for a more homogeneous and reproducible material was quickly identified.

Data generated from surrogate DCs used in methods such as h-CLAT (THP1 line), U-SENS (UP37 line), IL-8 Luc (validated, OECD TG 442E), and GARD (under validation, for inclusion in OECD TG 442E) [MUTZ-3 and SenzaCells (ATCC Depository PTA-123875)] address the same AOP (skin sensitization) event, namely DC activation. Each of the assays addresses a specific activation, U937 the expression of CD86, THP1 the expression of CD86 and CD54, IL-8 the production of IL-8, and as for the last one, the measurement of the expression of more than 200 mRNAs in a cell line derived from human myeloid leukemia, SenzaCell. While predictivity has been well demonstrated for all these assays, this is not the case for the progenitor or monocyte-derived DC models. Therefore, it is not easy to compare these DC models’ reproducibility and outstanding predictive performance with the data generated by surrogate DCs.

The models described in this review are useful for understanding the mechanisms of action of chemical molecules because they incorporate all the parameters measured by the OECD tests (C86, CD54, IL-8, mRNAs) and even more such as the Nrf2 pathway (Ade et al., 2009).

Indeed, depending on the mechanisms or products studied, these different DCs models are complementary to evaluate DC activation upon CS exposure. In this context, regarding the ability of BMDCs to activate T cells (TCs) *in vitro*, the BM-DCs model seems promising as it has recently been described to allow the classification of chemicals according to their allergenic potential (Battais et al., 2017; Huppert et al., 2018).

## Data Availability

The original contributions presented in the study are included in the article/supplementary material, further inquiries can be directed to the corresponding author.
